# Elderly Caregiving Quality Improvement: A Pilot Study of the Burdens of Vietnamese Caregivers in Taiwan

**DOI:** 10.3390/ijerph19106293

**Published:** 2022-05-22

**Authors:** Chang-Yu Wu, Yu-Ying Li, Maurice J. Lyver

**Affiliations:** 1 DOAE, National Taichung University of Science and Technology, No. 129 San-Ming Rd., Taichung 40401, Taiwan; s1310941038@gms.nutc.edu.tw; 2 Department of Nursing, National Taichung University of Science and Technology, No. 129 San-Ming Rd., Taichung 40401, Taiwan; s1310951009@nutc.edu.tw

**Keywords:** long-term care, foreign domestic worker, multilingual health education resources, thematic analysis

## Abstract

Taiwan is expected to reach super-aged status by 2026, leading to an increased demand for elderly caregiving services. Low local unemployment and a dwindling working-age population mean the island’s care system relies heavily on female foreign domestic workers (FDWs) from Southeast Asian neighbors such as Vietnam to satisfy labor shortages. Although suggested by anecdotal evidence, limited research has been conducted on the link between the shortfall in FDW qualifications, training, preparedness, and expertise and their employment stressors. Therefore, this study aims to assist FDWs by evaluating their stressors and helping them better understand health care delivery by (1) administering the Modified Caregiver Strain Index (MCSI) revised 2003 questionnaire, (2) performing semi-structured in-depth one-on-one interviews, (3) classifying interview results according to thematic analysis, and (4) using these themes to devise and deliver a 12-week multilingual health education teach-back program. Our results indicate that Vietnamese FDWs face specific challenges, including language barriers, homesickness, intensive physical and psychological work demands, stress adaptation, and occupational exposures. Despite yielding no significant improvements in caregiving strain, our intervention, conducted at the height of the COVID-19 pandemic, pinpoints and classifies areas of grave concern and proposes recommendations that can assist long-term care (LTC) stakeholders in understanding and overcoming their respective challenges, thereby improving the quality of elderly care.

## 1. Introduction

### 1.1. Foreign Domestic Workers (FDWs) in Taiwan

**Figure 1:**
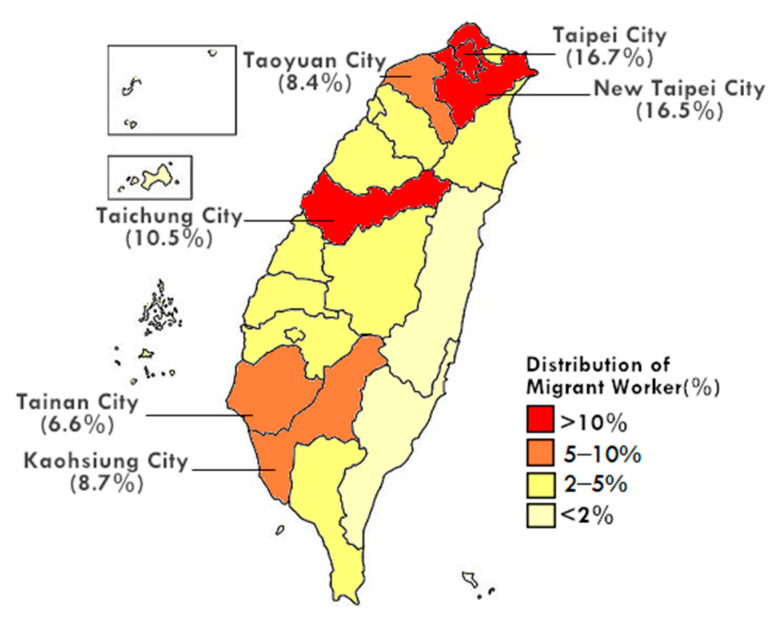
The percentage of migrant workers in social welfare by region in November 2021 [1]. Data source: adapted from https://english.mol.gov.tw/21004/21107/21113/lpsimplelist (accessed on 30 January 2022).

The healthcare setting in Taiwan relies heavily on FDWs such as direct care workers, home health care aides and personal care attendants. In 2021, the three metropolitan areas with the most migrant worker in the social welfare sector were Taipei, New Taipei, and Taichung City; see the data shown in Figure 1 [1,2].

**Figure 2:**
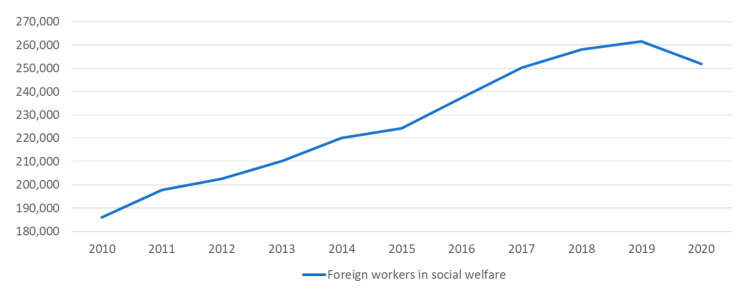
Foreign domestic workers from 2010 to 2020 [1]. Data source: adapted from https://english.mol.gov.tw/21004/21107/21113/lpsimplelist (accessed on 30 January 2022).

According to Liu (2012) [3], the majority of residents living in long-term care facilities in Taiwan possess moderate to severe disability levels. Lee, Kang and Liu (2022) [4] discussed the critical importance of long-term care in the Taiwanese context and highlighted that the problem is intensifying over time. While the ever-increasing demand for elderly care services seems irrefutable, how to address the labor forces issues remains understudied. Over the last decade, Taiwan has come to rely more heavily on migrant workers who make great contributions to the social welfare sector. This sector consists of five different worker classifications (caregivers, nursing assistants, outreach workers, domestic helpers, and domestic workers). From 2010 to 2019, there was a significant upward trend from approximately 190 thousand to 260 thousand foreign domestic workers. Figure 2 below shows the number of foreign workers in the social welfare sector from 2010 to 2020 in Taiwan.

Despite the increase in demand and the growing importance of FDWs, recent studies have raised concerns by showing that Taiwan’s FDWs and their burden had a negative relationship with the number of children, Chinese proficiency, and non-eldercare experience abroad, a positive relationship with education, and a non-monotonic relationship with marriage and, most importantly, elderly care experience [3,5]. Internationally, many aspects of the employment, training and retention of elderly caregivers have begun to receive greater scrutiny as a result of the mounting negative concerns [6,7,8]. Yet, in Taiwan, as many as 22% of FDWs had not received any elderly care training before they began their employment contracts. Furthermore, 77.9% of them claimed that the main difficulty was the language barrier, so they suffered trouble communicating [9].

**Figure 3:**
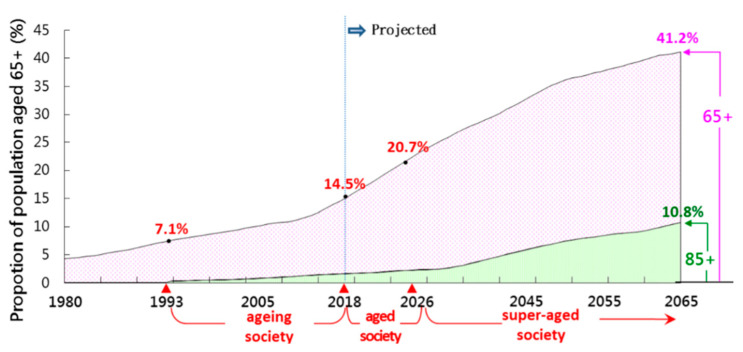
Population Projections for Taiwanese Elderly: 2018–2065 [10]. Data source: (adapted from https://www.ndc.gov.tw/en/cp.aspx?n=2e5dcb04c64512cc (accessed on 30 January 2022). (Source: National Development Council, 2018).

The situation is further exacerbated by the rapidly growing geriatric population in Taiwan. Figure 3 shows the alarming projected rate of increase in the elderly population. If the society’s aging rate exceeds 7%, it is deemed an “aging society”. Once the rate surpasses 14%, it is an “aged society”; beyond 21%, it is considered a “super-aged society”. Taiwan became an “aging society” in 1993 and by 2018 had already achieved the status of an “aged society”. The National Development Council has predicted that the rate will be over 21% (super-aged) by the year 2026 [10]. As Taiwan’s elderly population rises, the demand for elderly caregiving follows suit. To date, this caregiving role has almost exclusively fallen to female FDWs, and this trend is likely to continue. As a result, regulators must be mindful of the quality of care, safety, and cost-efficiency while providing adequate benefits and protections to these FDWs.

### 1.2. Linguistic Competence

Potter et al. (2016) [11] stated that organizational linguistic competence was the skill of all members of an organization to effectively and precisely relay information so that it is easily comprehensible by all. In Taiwan, strong Chinese language (Mandarin Chinese and Taiwanese Hokkien) skills should help FDWs overcome some of their work burdens as this assists them in communicating with people who speak these different dialects. FDWs who are not proficient in this regard are more likely to encounter more burdens [12]. In Singapore for example, FDWs who are not proficient in Chinese experienced a higher burden [13], and we therefore believe that Chinese skills (Chinese proficiency) will have a negative relationship to burden in the current context.

### 1.3. Domains of Learnings and Using Teach-Back as a Method of Health Education

Previous research has established that there are three domains in learning: cognitive (understanding), affective (attitudes), and psychomotor (motor skills) [11,14]. It is important to involve all three domains in health education, for example, a healthier lifestyle is in the cognitive domain, a coping mechanism is in the affective domain, and a ‘how to’ question is in the psychomotor domain.

The teach-back strategy is an intervention method used to help the health care provider confirm whether or not the patient or individual has understood correctly what they have been taught. Teach-back involves asking the acceptor to explain in their own words or via demonstration what the provider has just taught them. This is an ongoing process of asking the acceptor for feedback until one is confident that the acceptor has a full understanding of the information. The process can also ensure the acceptor has a better recall of the contents that they have been given. If there is any misunderstanding, the health care provider can correct or re-teach the skills. When using the teach-back technique, health care providers should ask open-ended questions to verify the acceptor’s understanding level [15,16]. It can help health care professionals to improve the safety of patients and caregivers, and it is also an effective communication method between health care workers with patients or caregivers. A 2020 systematic review by Talevski et al. (2020) [16] proved that effective communication can improve the safety and quality of health care. The same study also determined that the teach-back is a valuable evidence-based strategy used to evaluate the health education results in clinical settings and ensures acceptors can receive clear and concise information after the teach-back program [16].

### 1.4. Burdens

A recent Hong Kong scoping review indicated that the main stressors foreign domestic workers suffer include abuse, poor health services accessibility, social isolation, and financial hardships [17]. These issues, along with demanding working conditions, mean that their job does not pay well in terms of the demands placed on these workers for their effort and skill contribution [17]. In Singapore, foreign domestic workers face specific stressors which include financial problems, family separation, immigration challenges, culture shock, and language barriers [18]. Caregivers with a high burden experience decline both in terms of their physical and emotional health. According to Alves et al. (2019) [19], a high burden may cause burnout syndrome, which is characterized by three dimensions—emotional exhaustion (lack of energy or enthusiasm), depersonalization (being indifferent, impersonal), and a reduction of personal fulfillment. The authors note that burnout syndrome is likely to manifest itself in any of three ways—mentally (low self-esteem, fatigue, depression, stress, anxiety, and lack of concentration), physically (headache, pain, and gastrointestinal problems) or behaviorally (smoking, drinking, substance abuse, absenteeism and working negatively or ineffectively) and in-turn will affect the quality of care given and the caregiver’s health [19].

In addition to their social, psychological, and financial burdens, foreign caregivers also need to do physically strenuous tasks. Studies have found that many FDWs have poor mental health and suffer physically which in turn may cause them fatigue, sleep disturbance, and depression [13,17]. While mental and physical health are two seemingly separate domains, the two appear inextricably linked in that higher burdens compromise the caregiver’s physical and psychological health [20,21,22]. When caregivers have trouble relieving these types of stressors, it no doubt will affect their health and their work.

That FDWs in different regions are exposed to and suffer a range of stressors has been confirmed in the literature. Our research, however, argues that the problem is contextual, and the specific stressors vary across time and location. Thus, caregivers require specific knowledge and skills relevant to their specific needs in order to tackle their stress. Given the rapidly accelerating aging population in Taiwan and the resultant increased demand for caregivers, this study aims to evaluate FDWs’ current levels of stress using MCSI, perform semi-structured interviews with study participants, understand and classify the types of stressors encountered, and develop and implement a teach-back intervention to help caregivers cope with their work stress. The results may help the caregivers themselves, their instructors, relevant authorities and ultimately the elderly care recipients.

## 2. Methods

This research employed an action research design. Quantitative and qualitative data were collected from Vietnamese foreign caregivers who work in nursing care facilities via semi-structured interviews, thematic analysis, and the MCSI questionnaire. Inclusion criteria were as follows: (a) ability to use English or Chinese to communicate; (b) legal employment in a care facility and having worked for more than three months; (c) current employment as an FDW, including providing care to the elderly or those 65 years old and above; (d) age between 20 and 65 and agreement to participate in the study.

In our 12-week study, we identified foreign caregivers’ strains by conducting one-to-one interviews in the first two weeks. The semi-structured interview guide developed from the previous literature is presented in Table 1. Interviews were transcribed verbatim and classified into themes using thematic analysis.

Then, based on the results of our thematic analysis, we designed and (using a teach-back method) gave relevant health education including bedside teaching, wide-ranging discussions, listening and empathy over the remainder of the study period. Video clips and slides were used in our educational materials. These video clips and materials contained Vietnamese subtitles, and our bedside teaching included common caregiving skills such as tube feeding and positioning. Bedside teaching can be treated as part of motor learning. The participants learned from the motor act, sensory information, perception, and knowledge. The teaching program was aimed to help the foreign caregivers reduce their work stress so that their routine work could be carried out more easily. Listening and wide-ranging discussions were intended to give them moral support and improve their working conditions.

### 2.1. Recruitment and Participants

This study was initiated by the National Taichung University of Science and Technology, which is a vocational and technological university located in central Taiwan. The data collection period was from 1 July 2021 to 20 September 2021. Formal verbal invitations were extended to two different nursing facilities employing FDWs. We employed a purposive sampling technique and a total of 10 out of the 20 Vietnamese FDWs employed at these two facilities were deemed eligible and all agreed to participate in the study. Ethical approval was first obtained from the relevant institutional review board (IRB) and informed consent was received by all 10 participants prior to initiating research.

### 2.2. Using Modified Caregiver Strain Index (MCSI) Revised 2003

The Caregiver Strain Index (CSI), originally developed in 1983, was a tool used to quickly screen for caregivers’ strain in performing care. The CSI is a short and easily self-administered measurement scale [23]. There are 13 questions to measure strain related to care, which include: Physical, Social and Personal, Psychological, and Financial dimensions. Each ‘YES’ answer generates 1 point and each ’NO’ answer 0 points. The CSI displayed both high internal consistency and reliability and a reasonable Cronbach’s alpha of 0.86 [23,24].

However, because long-term caregivers were not comfortable with the dichotomous responses in the original CSI, the MCSI was developed in 2003. The questionnaire options are ‘Yes, on a regular basis’ for 2 points, ‘Yes, sometimes’ for 1 point and ‘No’ for 0 points [24,25]. The level of caregiver strain in the MCSI is categorized as low (0–8), moderate (9–18), and high (19–26) with a maximum score of 26 and a minimum of 0 [26]. The reliability coefficient for the MCSI (α = 0.90) is slightly higher than CSI (α = 0.86). The additional options on the Index provided an enhancement [24]. The index provides valuable insights for nursing workers to identify the stresses in caregivers and help them to improve those problems [27]. Therefore, the MCSI was chosen as the measurement instrument for our study. The Vietnamese version of the MCSI is included in Appendix A.

### 2.3. Thematic Analysis

Thematic analysis (TA) was chosen as the method to understand more deeply the caregivers’ stressors, behavior, and experiences. It is widely considered a powerful method for analyzing qualitative data. TA can build a broader construct in numerous social contexts due to its flexibility to generate new insights. The method also allows researchers to summarize and highlight the key data easily [28]. TA can analyze data either inductively (data-driven) or deductively (theory-driven) and it has been used in almost every conceivable field of scholarship, thus it suits the general qualitative research approach [29].

TA can narrow the qualitative analysis data depending on the researcher’s aims or goals. It is a method for describing data by selecting codes and constructing themes. TA can also be applied to study questions, designs, and sample sizes. It is generally agreed that TA is a flexible method to express different kinds of qualitative data from varied perspectives [28,29]. In order to carry out TA, Nowell et al. (2017) [30] described six main steps which are required to establish trustworthiness, including (i) data familiarization, (ii) initial coding, (iii) theme search, (iv) theme review, (v) theme definition and naming, and (vi) report production. To generate initial codes, it is necessary to read and familiarize the verbatim scripts back and forth, and carefully analyze the meaning in each section of the text, defining which sections are related to the topic and which are not, then letting the sentences give the initial concepts. This step requires the researcher to repeatedly revisit the data.

In our analysis, once the original data became saturated, coding began. Coding is a process of data reduction and simplification. In this phase, it was important to find interesting features in the data items that may lead to the formation of themes. Each code should have quite clear and demarcated boundaries, and not be interchangeable, redundant or overlap with other codes [31]. Braun and Clarke (2006) [32] suggested that care and attention be given to the entire data set and each data item must be given full consideration. For example, responses that seem to veer from a particular theme should not be dismissed outright as may in fact lend to the overall narrative [32,33].

In the next step, we identified themes by connecting portions of the caregiver’s experiences. DeSantis and Ugarriza (2000) [34] provided the following definitional guidelines for researchers,

*“A theme is an abstract entity that brings meaning and identity to a recurrent experience and its variant manifestations. As such, a theme captures and unifies the nature or basis of the experience into a meaningful whole”.*

Most of the time researchers will put several codes into a theme, and codes which are too vague or not relevant to the theme can be discarded [32,34].

Themes can be generated either inductively or deductively [35]. In this research, we employed an inductive approach by linking the data obtained to the themes identified. Inductive analysis is data-driven and avoids the use of established frameworks and preconceptions [32]. Derived themes should hopefully highlight important concepts which connect the data [34].

Once a set of themes had been devised, a review of the themes began. This required refinement and various changes. During this phase, researchers may find that some themes are insufficiently developed or divergent from the core theme. Each theme and its data should be meaningful, with a clear and identifiable distinction. According to Nowell et al. [30], a final review should be thorough and researchers should ask themselves: “Does each theme contain enough supporting data?” and “Are the data included coherent in supporting each theme?” After determining what aspect of the data has been captured, researchers can conduct a final analysis and provide a name for each theme. Lastly, once the researcher is confident that they have a fully established set of themes, report production may begin.

## 3. Results

### 3.1. Participants’ Characteristics

As Table 2 shows, the full sample included 10 participants. Of the 10 participants, all (100%) were female. The caregivers ranged in age from 20 to 59 (N = 10). All 10 (100%) of them were Vietnamese. In total, 7 (70%) of the caregivers had prior caregiving experience, and all 10 (100%) of them had some degree of prior caregiving training. The demographics of the participants are shown in more detail in Table 2.

### 3.2. The Modified Caregiver Strain Index (MCSI)

The strain level of all 10 participants was evaluated using the MCSI questionnaire and is shown in Table 3. In the pre-test, the average score was 5.3 out of a maximum of 26; the questions that received the higher scores were ‘Caregiving is inconvenient’ and ‘Caregiving is a physical strain’. In the midterm test, the average score had climbed to 10, and it highlighted the questions ‘Some behavior is upsetting’ and ‘There have been work adjustments’. Following the post-test, the average score had increased yet again to 12.1 with the higher scores on the questions ‘There have been work adjustments’ and ‘I feel completely overwhelmed’.

According to the result of the MCSI questionnaire, the scores increased chronologically from the first week, the sixth week, and the twelfth week. The study was conducted during the COVID-19 pandemic. On 11 June 2021, with a heightened level 3 epidemic alert, visa processing was halted and foreign national entry into Taiwan was suspended. An overall lack of personnel and the inability to secure new hires combined with overwhelming workloads may have caused increasingly higher stress levels for caregivers over the study period. That their stress level increased in such a short time period was and is a serious cause for concern. The foreign caregivers who participated in our study possessed adequate caring skills and had prior caregiving experiences. With the unexpected factors affecting our study, teach-back intervention did not appear to have any significant improvement in MCSI.

The results of our study showed low (0–8) to moderate (9–18) levels of caregiving stress. However, this research which was carried out over a 3-month period has captured a significant and worrying spike in caregiver strain as measured by the MCSI. We identified several factors that the FDWs claimed sometimes affected them. These factors were the care recipients’ upsetting behavior, work adjustments, financial strain, overwhelming job demands, and difficulty in managing time. The foreign caregivers in our study reported that some of the care recipients’ behavior was upsetting (e.g., incontinence, trouble remembering things, or being accused of taking things). These behaviors commonly happen among people who suffer from cognitive disorders. They also claimed that there have been working adjustments. With the lack of labor (due to COVID-19 travel restrictions), they sometimes had to give up their free time and take on extra caregiving duties. Financial strain also seemed to spike over the period of study possibly as a result of the negative economic spillovers arising from the pandemic. They also mentioned that there have been many more demands on their time (e.g., their family members abroad need to be more supportive). Finally, MCSI results indicate a significant increase in the question—they feel completely overwhelmed. These combined increases in caregivers’ strain have quite possibly hampered their quality of life.

### 3.3. Thematic Analysis Major Themes

Our thematic analysis revealed five major themes, with a total of 18 subthemes as shown in Table 4. The major themes are ‘Language Communication’, ’Work Burden and Job Strain’, ‘FDWs’ Responsibility and Risk’, ‘Stress and Resilience’, and ‘Homesickness among Expatriates’.

#### 3.3.1. Theme 1: Language Communication

1. Struggling in New Job in a Foreign Country

Launching a caregiving career can be daunting in a foreign country. Before the Vietnamese caregivers came to Taiwan, most had only received brief training in the Chinese language. The training time was short; they had only taken (2–6) month-long courses. In the early stage of starting their job, they could hardly use Chinese in daily communication and did not have basic listening and speaking skills. Using Chinese to communicate was one of their major challenges.

*“When we came to Taiwan, we couldn’t communicate in Chinese at first”.*

(P01)

*“When we first came to Taiwan, we could not understand Chinese very well. Although we have studied it in Vietnam, we found it was different from normal daily conversation”.*

(P08)

In addition, the Chinese courses taken in Vietnam were taught by Vietnamese teachers and they had strong Vietnamese accents. The care recipients’ accent is vastly different from the foreign domestic workers who learned Chinese in Vietnam. The FDWs could not coherently use Chinese upon arrival in Taiwan and speaking Chinese with a Vietnamese accent meant that they could not be understood easily.

*“Before I came to Taiwan, I had learned Chinese in Vietnam. In the beginning, people couldn’t understand what I talked about because of my Vietnamese accent”.*

(P06)

2. Communication Difficulties and Its Impact

Poor Chinese competence may cause communication issues in the workplace, such as (i) being unable to understand what tasks they are responsible for, (ii) being unable to use Chinese coherently, (iii) not being able to use Chinese to express their thoughts. Due to their (FDWs) strong accent, Taiwanese people may not understand clearly what they say.

*“At work, some people cannot understand what I mean. It is hard for me to speak Chinese without an accent. When I speak Taiwanese (a Chinese dialect), the situation is even worse”.*

(P07)

*“We can understand what people are saying to us, but we still cannot express ourselves in Chinese clearly”.*

(P08)

3. Consequences of Poor Communication

If the FDWs suffer from poor communication ability, it will have a negative impact on their work and life. For instance, if they do not understand the language used in their workplace, it could lead them to have to work extra time, create a heavier workload, and increase their working hours. Moreover, poor communication in Chinese will cause them to be misunderstood or distort their actual meaning which causes negative impacts on their work.

*“The communication problem did affect my job … a colleague asked me to take some items, but I got the wrong ones because I did not understand exactly what they meant”.*

(P06)

*“I got into trouble communicating at work … and it has caused many negative impacts. It is hard for me to deal with the communication problem. If it happens, all of my schedules will be delayed. Commutation problems have made the job even more difficult for me”.*

(P07)

4. Difficult to Communicate in Taiwanese Hokkien

In spite of the widespread use of Mandarin Chinese, they also need to speak Hokkien which is the most commonly spoken language among elderly people in Taiwan. In the FDWs’ training program, they did not have any exposure to the Hokkien language, and they were not even aware of the existence of Hokkien. However, the residents that live in the care facilities mainly speak Hokkien. Thus, the FDWs faced trouble communicating in both Mandarin Chinese and Taiwanese Hokkien.

*“I have only learned Chinese (Mandarin Chinese), so I can’t communicate in Taiwanese (a Chinese dialect); it is so difficult. The elderly here speak Taiwanese, but I can’t understand what they are talking about”.*

(P03)

*“I had never heard people speak Taiwanese until I arrived in Taiwan. We didn’t learn it in Vietnam”.*

(P10)

#### 3.3.2. Theme 2: Work Burden and Job Strain

1. Work Responsibility of Caregiver

There is a fixed routine at their caregiving work—every day is the same. Their main duties include lifting and transferring the elderly, changing their diapers, replenishing amenities, helping the elderly take baths, feeding them, and performing nasogastric tube feeding. Through practice, they have gotten used to their job which is highly repetitive. From time to time however, they also need to handle medical emergencies.

*“… We look after the residents, and sometimes we do the additional work they request. If there is no nurse around, and residents have something urgent, we have to deal with it”.*

(P02)

*“I change their body position, change their diapers, replenish amenities, help them to take baths, feed them, and do nasogastric-tube-feeding for residents. I also help them to do the laundry, move them from bed to a wheelchair, and clean the toilets. There are so many chores to do”.*

(P04)

2. Ordeals at Work

The care recipients may insist on their thoughts/ideas and fail to follow the instructions from the FDWs. They may also grow restless, irritable or have cognitive impairment due to their illnesses. The foreign domestic workers sometimes are verbally bullied by their care recipients and that will make their work more difficult. Besides, FDWs have to work shifts and must hand over to the next shift. Having quarrels with colleagues or the person who works the next shift is also an ordeal at work.

*“Sometimes we ask the residents to do something they don’t want to do; they will get irritated. Due to their illness, the elderly remains annoyed and cannot calm down. I will feel irritated! This is not an easy job”.*

(P01)

*“There are problems between my colleagues … I get incomplete work tasks from my colleagues sometimes”.*

(P05)

*“Some of the elderly don’t want to go to the toilet frequently, so they decide not to drink any water. I will ask them to drink some, but no matter how hard I try, they refuse”.*

(P07)

*“It is impossible to handle each of them at the same time. Some of the elderly have a bad temper. They will scold us. And it is annoying if they murmur during the break time”.*

(P09)

3. Difficult Clients

Some care recipients demand more effort which is a huge challenge for caregivers. For instance, care recipients using wheelchairs are at a high risk of falls. People who try to move on their own may fall if the FDWs do not take precautionary measures. Besides, if the FDWs are unfamiliar with the care recipients who have recently moved to the care facility, the FDWs may commit medical negligence or unwittingly cause other problems. Additionally, people with dementia often have symptoms of unconsciousness or forgetfulness. They will forget what the caregiver has done for them and then they will make complaints to their family members. The care recipients’ occasional aggressive behavior may cause injury to the caregivers or other people. Moreover, those people who have lost their ability to speak out are not able to precisely let the caregiver understand their needs. The above-mentioned issues put severe pressure on the caregivers.

*“It is more stressful to take care of the residents who are in wheelchairs … they may fall down without any notice. We are worried and have to prevent falls. It really makes me stressed”.*

(P01)

*“The elderly will be harder to take care of if they have just come here recently because we know nothing about them”.*

(P02)

*“We have done things to help the residents, but they forgot, and they told their family members that we are unwilling to help them. It causes misunderstandings. They say something rude, too”.*

(P03)

*“The elderly who suffer from dementia may sometimes hit others. We are not afraid of being hurt ourselves. Instead, we are worried that the other elderly residents may get hurt”.*

(P06)

*“The residents keep calling my name, but they cannot remember why they asked me to come. They immediately forget what they said… they request us to do the same thing again and again”.*

(P10)

4. Physical Burden

Due to COVID-19, it is hard to hire additional FDWs. This situation has resulted in care facilities facing manpower shortages. Thus, the FDWs have had to bear more workload which makes them physically and mentally tired. Their job may cause occupational injury as well, such as sore muscles and back pain. Under this condition, the caregivers may want to change their workplace or even run away.

*“We are more understaffed now (in the COVID-19 pandemic). We don’t have enough caregivers here, so we need to do more tasks. It makes me a little bit tired”.*

(P03)

*“Changing the elderly residents’ position is tiring for me. My shoulder hurts. If I lift something heavy, my shoulder will be more painful”.*

(P04)

*“I feel tired of my job, and I want to get a new one. I need to walk around very frequently, and my legs hurt”*

(P10)

#### 3.3.3. Theme 3: FDWs’ Responsibility and Risk

1. Fear of Blame or Punishment

As a caregiver, one must take responsibility for their caring job, especially for the elderly care recipients’ safety. At work, caregivers will do their best to provide the best possible care. However, if accidents occur due to their carelessness, caregivers need to report the incident to the care recipients’ family members and they might be punished or blamed by them. Though they do not have to directly contact the care recipient’s family members, the supervisors and nurses will scold the caregivers. It causes emotional stresses for caregivers such as self-blame, worry and depression.

*“We are responsible for the patients’ safety. If they fall, we will be punished and have to notify their family immediately”.*

(P01)

*“We will need to tell their family and explain what happened. Although the nurse will deal with it, we think we also need to take responsibility for it. We will blame ourselves because we have not taken good care of them and caused them to get hurt. If any accidents happen, we will feel very sorry”.*

(P06)

*“I hope that the families and supervisors can be more compassionate. We have a lot of work to do, and it is hard to do everything perfectly”.*

(P09)

2. Supervisors and Care Recipients’ Families

Caregivers will sometimes need to talk to the elderly care recipients’ family members. Their family members may have different requirements and expectations of care, which sometimes may be unreasonable. Even if the caregivers have tried their best to take care of them, they would be criticized by the residents’ relatives or even scolded by their supervisors. Occasionally the elderly care recipients do not ask the caregivers directly, they talk to the supervisor or their family members to send their request instead. This behavior will often lead to the caregiver not being able to immediately deal with the problems they have encountered, then they would be scolded by their supervisor. This situation can cause the caregiver to suffer negative emotions as well.

*“Some of the families of the elderly are picky. For example, the hairdresser only comes in at a fixed time. The elderly cannot get their haircut whenever they want. Their families are always complaining about the length of their hair. Their families can complain about everything!”*

(P07)

*“Some families of the elderly have a world of requirements. For example, skin problems are common and they are usually dissatisfied about this. They think the diapers must be cleaned and be changed more often. We follow their instructions, but they still make complaints. Some elderly lie in bed for a long time, so their skin may be in bad condition; if we move them, they will get a scrape and the family will scold us”*

(P09)

*“Their family members will scold us if they find any wounds. Some elderly will even complain to their family members instead of telling us directly. It makes us stressed because the family would complain to the head nurse and other nurses”.*

(P10)

3. Occupational Exposure to Unknown Sources

The caregivers have to be in very close contact with residents at work. Although they wear masks, put on gloves, and regularly wash their hands to protect themselves, they are still exposed to contagious diseases. Colds and parasites are major concerns and scabies is common. Sometimes people are infected with something that might not show any symptoms at first, yet it is still transmissible. Before the symptoms can be seen, the caregivers have already had prolonged contact with those people. Occupational exposure to unknown sources causes worries and stress.

*“There is one thing that I worry about and that is if the resident is sick, but we don’t notice it immediately. We work with them closely and even live together. It is easy to get infected in our daily life, it is the most stressful thing in my work”.*

(P01)

*“We have been infected by the residents before. Sometimes the residents feel itchy under their skin or have skin problems that are contagious. Or when the weather is bad, the residents may catch colds and then we may be infected too”.*

(P05)

#### 3.3.4. Theme 4: Stress and Resilience

1. Emotions and Feelings

Trouble communicating in a foreign language will lead FDWs to feel stressed and uneasy. If they cannot use the Chinese language fluently, they may feel helpless because they are not able to express themselves. Moreover, if they are unable to do their tasks at work, they may be scolded by their colleagues, residents, and the families of the residents. FDWs will feel frustrated and generate negative emotions toward them.

*“It makes me stressed if I can’t understand what the work assignments are. I don’t know what I should do at that moment”.*

(P01)

*“If we were unfamiliar with our job routine after working here for a while, the colleagues who lead us would blame us. I feel stressed when this happens”.*

(P09)

*“I feel very stressed when I cannot understand other people’s meanings or … I cannot handle my job well … the elderly also scold us too. I couldn’t understand what I am supposed to do at work, I even couldn’t understand what the elderly scold us, it is very stressful”.*

(P10)

2. Way to Relieve Stresses

Elderly caregivers have various ways to relieve their stress. For example, sharing their story with their family members or friends, sleeping more, shopping, or drinking beverages will make them feel better.

*“I go shopping when I feel tired from work, sometimes I enjoy playing on my cell phone, and hanging out with my friends. It will make me feel relaxed”.*

(P02)

*“I will share stories with my friends, talk to them about something interesting, and tease them. They will make me feel happy”.*

(P04)

*“I take a good rest, lie on my bed and sleep more when I face frustrations at work or suffer from fatigue. Going shopping after work can also make me happier”.*

(P05)

*“When I am upset, I will get something to drink, or listen to music”.*

(P07)

3. Dealing with Deaths

The caregivers may face the residents’ death. Some foreign caregivers have not worked in healthcare-related jobs before, and this type of experience may be a first for them. When they see deceased persons, they feel worried, scared, stressed, and even shocked. How to develop their psychological adaptability in dealing with the emotions related to death is an important lesson to learn.

*“I have seen some deaths before. I felt stressed when I faced this kind of situation. Thus, I am worried about my family in Vietnam, and I miss them very much”.*

(P04)

*“I felt very nervous and scared to look after the dying seniors. I worked in a factory in Vietnam before, so I had never encountered this kind of problem”.*

(P08)

*“When I take care of the elderly who are dying, I am terrified. I am afraid of ghosts. Because of this reason, I hate to work the night shift especially in the Ghost Month. I would even bring a garlic in my pocket”.*

(P09)

#### 3.3.5. Theme 5: Homesickness among Expatriates

1. Expatriates Worried about Their Families

The FDWs are residing in Taiwan rather than their native country, Vietnam. They come to Taiwan to work, and their job is related to elderly health care and dealing with sick, immobile, and infirmed residents. This may cause worries for their own family members in their hometown, especially when they see that some of the seniors are ill. Their (FDWs) minds are filled with concerns and multiple questions: Who will come and help if both my parents get sick? Who can provide help if my family members have medical problems? My family needs my help, but I cannot be there. If my family members get sick or even pass away, I cannot be there with them. The above situations may make the caregivers more worried at work.

*“I have started to worry about the elderly in my family since I began working here. My grandmother is even over 90 years old. I am afraid that I can’t keep them (my family members) company when they are sick”.*

(P01)

*“Since I began working in Taiwan, I have started to worry about my family. I cannot travel back to my home country whenever I want. I am worried about them because I cannot see them face to face”.*

(P06)

2. Missing Home

Most of the FDWs have come to Taiwan alone and they normally do not travel back home until their work permit has expired. They cannot meet their families or friends every day, and they always miss them. In that way, they strengthen their relationship by staying connected on social media via making a phone or video call. Moreover, the FDWs who are married tend to suffer more intense homesickness. They miss their children and their loved ones very much.

*“Although I love my job in Taiwan, I always feel homesick. At this moment, I am eager to go back to Vietnam and work there”.*

(P02)

*“I miss my children, family, and friends very much, but I can’t go back”.*

(P07)

*“I’m married, and my husband is in Vietnam. Even though I miss him, I cannot meet him”.*

(P08)

3. Returning Home

Most of the FDWs plan to stay in Taiwan until their work permit has expired unless something urgent happens at home, in which case they can travel back temporarily. However, with the COVID-19 pandemic, international flights have declined, and everyone needs to follow strict and constantly changing travel restrictions. Although the caregiver can gain approval to return home, it is not easy to travel back to Taiwan due to strict quarantine restrictions. The additional fees such as quarantine fees, need to be paid for by the FDWs. Coupled with the expensive flight tickets, it would be a financial burden for the FDWs. In this situation, it reduces the likelihood and willingness of caregivers to travel back to their hometowns.

*“I must be working here for three years then I can go back to my country. With the regulations in Vietnam, I cannot buy a flight ticket whenever I want”.*

(P02)

*“If the COVID-19 situation improves, I can go back, but I still need to quarantine so it will take much time. People who want to travel back to Vietnam need to get vaccinated and quarantine, and when I travel back to Taiwan I need to quarantine again. The fee for quarantine is expensive and I also need to pay for flight tickets. It is very expensive”.*

(P05)

*“Because of COVID-19, there is only one flight per week to Vietnam, and rarely a flight back to Taiwan”.*

(P10)

4. Support from Family and Friends

FDWs are far away from home. Besides the salary and working conditions, the support from their own family and friends plays an important role in their decision to have work in Taiwan. In fact, most of their family members and friends oppose their decision to work in Taiwan. More recently, the salaries in Vietnam have gradually increased and are currently almost on par with those in Taiwan. In many cases, their family members hope that the caregivers can return home to Vietnam and work there. Still, most of their family members and friends try and be supportive. Although they are living in a different country, family members and friends give the caregivers spiritual encouragement.

*“In the past, my family members and my friends thought working in Taiwan was better than in Vietnam. Both my husband and my younger brother work here. However, now, my parents want me to go back to Vietnam and stay with my sister after the pandemic. Her salary is pretty good in Vietnam now”.*

(P06)

*“My family would like me to go back to Vietnam, but they still support me to stay in Taiwan”.*

(P08)

*“My friends in Vietnam hope I will go back. I only get minimum wage in this job. My classmates have their own homes and cars in Vietnam, but I have nothing here (in Taiwan)”.*

(P09)

## 4. Discussion

The multifaceted and complex nature of foreign caregiving has been shown in our thematic analysis which revealed the major stressors experienced by FDWs in the Taiwanese context.

Principally, communication skill is deemed most vital. Poor communication could jeopardize the FDWs’ interpersonal relationships with their colleagues, care recipients and their family members. Language-related communication barriers with the care recipient or care recipient’s family can be described as risk factors for FDW’s mental health [36,37]. Our thematic analysis revealed that employers, supervisors, and colleagues all contribute to the FDW’s mental health status, whether it be positive or negative. Thus, these caregivers need knowledge and skills relevant to their specific needs to tackle their stress.

In the United States, linguistic competency has been formally addressed by standard-setting and accrediting bodies including The Joint Commission (TJC). TJC, a non-profit tax-exempt 501(c) organization has certified more than 22,000 US health care organizations and programs. Its mission is: “to continuously improve health care for the public, in collaboration with other stakeholders, by evaluating health care organizations and inspiring them to excel in providing safe and effective care of the highest quality and value [38]“.

Thus, it is suggested that legislation related to long-term care be expanded to cover the linguistic competency of FDWs. We suggest that language training programs designed to improve FDWs’ linguistic competency should be offered in both Mandarin and Taiwanese prior to departure from their home country and incorporate sufficient continuing education post-arrival. Legislative reform may provide a solution to the problem in the long term, but in the short term, we recommend that translation applications can be useful in overcoming language barriers. Google Translate is the most common and convenient app for translation. The app is free and supports over 100 languages worldwide, but to our knowledge, none of the translation apps support Southern Min (or Taiwanese Hokkien). A translation app may help nursing facility staff to complete their work tasks more easily, but it is still not accurate enough to be trustworthy in translating medical terminology [39,40]. These apps could still serve as a free initial mode of translation between Vietnamese and Mandarin and vice versa. However, it should be noted that currently the use of smartphones by FDWs is generally frowned upon, especially within nursing facilities.

Another albeit more expensive short-term solution would be to incorporate the use of smart AI home assistant devices in elderly care homes employing FDWs. Although this method would be costlier and involve data security and other ethical issues [41], the list of potential benefits is long and includes: translation and interpretation, controlling other devices (lights), playing music, reading the news, making and receiving voice calls (programmable to limit certain users), collecting data, recording, and linking with other health monitoring devices (smartwatches, Fitbit) [42]. The more expensive varieties offer video control TVs, allow for video calling, perform virtual assistant tasks, can be controlled externally, and are more programmable. While the use of such devices has to be carefully considered by both families and facilities, they could provide increased peace of mind for loved ones and help ease the pressing issue of communication problems.

Secondly, prior studies and our thematic analysis have shown that FDWs have received inadequate preparation for some of their work tasks. Some FDWs have even been asked to provide nursing tasks without prior preparation [43]. According to Article 18 of the Taiwanese “Long-Term Care Services Act”:

*“All long-term care personnel should receive appropriate and adequate training programs. The training, continuing education, and on-the-job training programs for long-term care personnel shall consider the differences among regions, ethnic groups, genders, specific illnesses and care experiences”.*

[44]

In Taiwan, FDWs are supposedly required to complete a 96-h training program, a 50-h core knowledge lecture, 8 h of practical training, 30 h of clinical practicum, and two hours of evaluation [44]. However, recent news reports have suggested that authorities in the Ministry of Labor do not always verify that these training requirements have been fulfilled. Moreover, barely any FDWs have enrolled in enrichment programs that aim to improve their caregiving skills.

In this respect, the legislation is in place; however, adequate implementation and enforcement are critical. Previous research has already demonstrated that appropriate legislation can improve the health condition of FDWs [18]. Thus, we believe that the role of continuing education in the form of these types of enrichment programs should fall to geriatric/LTC nurses.

Thirdly, caregiving is a job that demands considerable amounts of physical work. Caregivers need to assist the elderly in their daily activities such as bathing, toileting, feeding, body positioning, getting in and out of bed, and dealing with incontinence. Long working hours may require their physical and psychological efforts, which in turn may cause muscle strain, back pain, or other chronic illness [45,46]. Their physical burden may also cause negative affectivity [43]. Our MCSI results display an exponential rise in the questionnaire item related to feeling completely overwhelmed in their current caregiving position. With the unprecedented COVID-19 pandemic, working conditions have worsened; it is now harder to hire or replace caregivers so existing FDWs have to bear more of the workload, and they are showing signs of strain and burnout. Our interviews confirmed that the increasing score on the MCSI was partly due to the added challenges stemming from the pandemic. This unforeseen crisis has in a relatively short period of time significantly damaged their quality of life. Additionally, people working in these types of healthcare-related jobs are at high risk of contracting contagious diseases. The occupational exposure that caregivers suffer includes influenza, parasites, scabies, tuberculosis (TB), hepatitis virus infection, and blood-borne diseases (HIV and hepatitis viruses) [47]. Possessing adequate knowledge of the diseases and symptoms the elderly care recipients face may reduce the level of caregiver anxiety and is another reason to engage geriatric/LTC nurses as trainers, supervisors, and mentors to help reduce the strain on FDWs.

Lastly, we focus on homesickness which is related to FDWs’ mental health. Mok (2019) [48] revealed that the hardships among FDWs include loneliness and homesickness, and she noted that these are mostly identified in female FDWs [48]. A 2004 study by Iyer et al. (2004) [49] stated that the most prevalent issue related to caregivers’ health that emerged was homesickness. Similar to our results, it was indicated that the FDWs who were married tend to suffer more intense homesickness. Our research participants told us that they missed their children and their loved ones very much. Homesickness can develop fear and anxiety of expatriation which in turn could significantly decrease people’s psychological well-being. Chronic fear may damage the immune, the nervous, and the cardiovascular systems the most [50]. Moreover, caregivers who originate from less developed countries may have higher psychological and physical strain which can inhibit these expatriate’s ability to cope with their stress [51].

Some of our research respondents added that the higher salary was the reason they chose to work in Taiwan and this fact has been confirmed in recent reports [52,53]. However, our interviewees said that the salaries in their home country are now catching up with Taiwan’s and under this situation, FDWs are more likely to return to work in their home country.

### Study Limitations

This study has a number of limitations which highlight the need for continued research. First of all, regarding the sample size, the number of participant caregivers equaled 10 in total. Although potentially adequate for small- and medium-scale studies according to Braun and Clarke, 2013 [54], and echoed by Smith, 2015 [55], this is one area where future researchers can expand their attention. At present, FDWs in our research setting hail from Vietnam, Indonesia, and the Philippines. Future research could expand the sample size and look at the differences among nationalities by employing other qualitative methods including focus groups or qualitative surveys. It should be noted also that more than 99% of all FDWs in Taiwan are female, leaving open the possibility for future study into the gender differences in caregiver burdens. Secondly, this study looks only at the burdens of Vietnamese caregivers in the Taiwanese context. We recommend that similar research be carried out in other locals where FDWs comprise an important and significant proportion of elderly caregivers. These could include but are not limited to countries such as Japan, Singapore, Macau, and Hong Kong.

## 5. Conclusions

This research set out to administer the MCSI on Vietnamese caregivers, conduct in-depth semi-structured interviews with all participants and then via thematic analysis, classify interview results before creating and delivering an extended health education teach-back program to participating caregivers.

Our study results highlighted that FDWs face specific challenges such as language barriers, inadequate training, intensive physical and psychological work demands, pressures from supervisors and care recipients’ family members, occupational exposures, and homesickness. Their caregiving strain scores assessed by the MCSI were 5.3, 10, and 12.1 (low to moderate stain level) in the first, the sixth, and the twelfth week of our study, respectively.

Given the unprecedented growth rates in the aged population here in Taiwan and abroad (America, Europe, and Asia) and the rise in the use of FDWs to care for these individuals, a number of implications can be drawn based on our findings. First, we assert that legislation needs to be enacted to reflect the current situation and thereby protect all stakeholders. Second, the use of technology in the form of translation applications and if possible, the implementation of smart AI home assistant devices should help overcome some of the specific language communication issues encountered as well as provide other benefits. Furthermore, competency improvement programs with multilingual materials may improve caregivers’ psychological adaptability, however, careful attention needs to be paid to the complicated and multifaceted nature of their caregiving role. When devising learning programs, we suggest targeting caregivers’ needs by understanding their previous healthcare training, caregiving knowledge, and prior work experiences. Finally, we suggest that the authorities provide adequate training programs to FDWs via geriatric/LTC nurses. Geriatric nurses can improve health care by advocating for a pleasant working environment and empowering FDWs with adequate health literacy. Furthermore, whenever possible, the system needs LTC nurses to plan programs adopted in the FDWs’ native languages.

The majority of FDWs in Taiwan are female and come from different regions in Southeast Asia. Most previously published research studies about these workers focus on the female FDWs’ race, class, and gender discrimination experiences. However, their health condition has received less attention. Female FDWs suffer more unfavorable working conditions and associated health problems than their male counterparts including verbal, physical, and sexual abuse, musculoskeletal strain, mental illness, and infectious diseases and combined they increase burden and work stress [46]. With the increases in FDWs globally and the aging society, these issues are deserving of heightened research attention and therefore we hope that this study increases our understanding of female FDWs burdens.

At this time of the global pandemic, there may be more risk factors for caregivers’ mental health. Providing related educational programs to FDWs is urgently required in Taiwan as critically insufficient staffing has created a severely strained work environment amidst the COVID-19 pandemic. We hope that these findings and recommendations can help LTC personnel and other stakeholders including lawmakers, caregiver institutions, and caregiver trainers understand the challenges and needs we presently face and move towards improving the current situation for the betterment of our beloved elderly.

## Figures and Tables

**Table 1:** Semi-structured interview guide.

Outline
“What do you usually do for your care recipient?”
“How did you learn to provide care for your care recipient?”
” What difficulties did you face when providing care?”
“How do you overcome the difficulties you have faced?”

**Table 2:** Demographics of participants.

	Participants	
	N (%)	Mean (SD)
Gender		
Female	10 (100)	
Age (years)		37.50 ± 8.23
20–29	1 (10)	
30–39	6 (60)	
40–49	2 (20)	
50–59	1 (10)	
Nationality		
Vietnamese	10 (100)	
Highest education level		
Secondary (Junior High School)	2 (20)	
High school	6 (60)	
University or college	2 (20)	
Marital status		
Married	8 (80)	
Single	2 (20)	
Number of children		1.00 ± 0.82
0	3 (30)	
1	4 (40)	
2	3 (30)	
Language for communication		
Chinese	10 (100)	
Taiwanese	8 (80)	
English	3 (30)	
Vietnamese	10 (100)	
Caregiving experience in their current position (years)		5.80 ± 2.83
<1	1 (10)	
1–2	1 (10)	
3–4	1 (10)	
5–10	7 (70)	
Daily hours spent for caregiving (hours)		13.30 ± 2.53
6–11	2 (20)	
12–17	8 (80)	
Prior caregiving experience		
Yes	7 (70)	
No	3 (30)	
Prior caregiving training		
Yes	10 (100)	
No	0 (0)	

**Table 3:** The modified caregiver strain index of participants.

		Average Score		
		Pre-Test (First Week)	Mid-Test (6 Week)	Post-Test (12 Week)
Physical	1. My sleep is disturbed	0.7	1	0.9
	2. Caregiving is inconvenient	0.9	0.3	0.6
	3. Caregiving is a physical strain	0.9	0.6	1.0
Social/personal	4. Caregiving is confining	0	0.8	0.9
	5. There have been family adjustments	0.1	0.6	0.5
	6. There have been changes in personal plans	0	0.8	0.6
	7. There have been other demands on my time	0.4	0.9	1.1
	8. There have been work adjustments	0.2	1.1	1.4
Psychological	9. There have been emotional adjustments	0.5	0.7	1.0
	10. Some behavior is upsetting	0.2	1.1	1.1
	11. It is upsetting to find the person I care for has changed so much from his/her former self	0.7	0.6	0.6
	12. I feel completely overwhelmed	0.3	0.7	1.3
Financial	13. Caregiving is a financial strain	0.4	0.8	1.1
	Total (maximum 26) low (0–8), moderate (9–18), and high (19–26)	5.3	10	12.1

**Table 4:** Coding structure.

Themes	Subthemes
Language Communication	(1) Struggling in New Job in a Foreign Country (2) Communication Difficulties and Its Impact (3) Consequences of Poor Communication (4) Difficult to Communicate in Taiwanese Hokkien
2. Work Burden and Job Strain	(1) Work Responsibility of Caregiver (2) Ordeals at Work (3) Difficult Clients (4) Physical Burden
3. FDWs’ Responsibility and Risk	(1) Fear of Blame and Punishment (2) Supervisors & Care Recipients’ Families (3) Occupational Exposure to Unknown Sources
4. Stress and Resilience	(1) Emotions and Feelings (2) Way to Relieve Stresses (3) Dealing with Deaths
5. Homesickness among expatriates	(1) Expatriates Worried about Their Family (2) Missing Home (3) Returning Home (4) Support from Family and Friends

**Figure A1:**
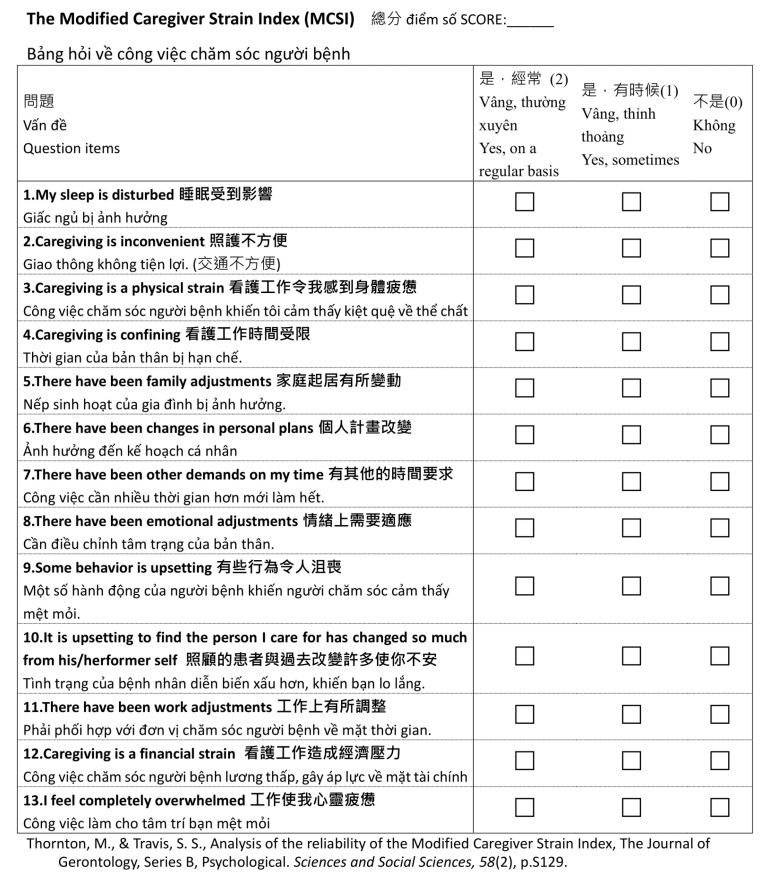
Vietnamese Version of Modified Caregiver Strain Index Revised 2003 [24].

## Data Availability

The data presented in this study are available on request from the corresponding author.

## Appendix A

Vietnamese Version of Modified Caregiver Strain Index Revised 2003.
